# Application of family-based tests of association for rare variants to pathways

**DOI:** 10.1186/1753-6561-8-S1-S105

**Published:** 2014-06-17

**Authors:** Brian Greco, Alexander Luedtke, Allison Hainline, Carolina Alvarez, Andrew Beck, Nathan L Tintle

**Affiliations:** 1 Department of Mathematics and Statistics, Grinnell College, 1115 8th Ave, Grinnell, IA 50112, USA; 2 Division of Biostatistics, UC Berkeley, 367 Evans Hall, Berkeley, CA 94720, USA; 3 Department of Statistics, Baylor University, 1511 S. 5th St, Waco, TX 76798, USA; 4 Department of Biostatistics, Florida International University, 11200 SW 8th St., Miami, FL 33199, USA; 5 Department of Mathematics, Loyola University Chicago, 1052 W Loyola Ave, Chicago, IL 60660, USA; 6 Department of Mathematics, Statistics and Computer Science, 498 4th Ave. NE, Dordt College, Sioux Center, IA 51250, USA

## Abstract

Pathway analysis approaches for sequence data typically either operate in a single stage (all variants within all genes in the pathway are combined into a single, very large set of variants that can then be analyzed using standard "gene-based" test statistics) or in 2-stages (gene-based *p *values are computed for all genes in the pathway, and then the gene-based *p *values are combined into a single pathway *p *value). To date, little consideration has been given to the performance of gene-based tests (typically designed for a smaller number of single-nucleotide variants [SNVs]) when the number of SNVs in the gene or in the pathway is very large and the genotypes come from sequence data organized in large pedigrees. We consider recently proposed gene-based tests for rare variants from complex pedigrees that test for association between a large set of SNVs and a qualitative phenotype of interest (1-stage analyses) as well as 2-stage approaches. We find that many of these methods show inflated type I errors when the number of SNVs in the gene or the pathway is large (>200 SNVs) and when using standard approaches to estimate the genotype covariance matrix. Alternative methods are needed when testing very large sets of SNVs in 1-stage approaches.

## Background

Until recently, the majority of methodological approaches for the analysis of common, single-nucleotide variants (SNVs; common SNVs have minor allele frequency of at least 5%) involved analysis of microarrays using either single-marker or multiple-marker approaches, with single-marker approaches, by far the more common of the 2 approaches in practice. Typically, large genome-wide association studies will conduct hundreds of thousands (or millions) of single-marker analyses. Although less common in practice, considerable methodological development has taken place in the area of multiple-marker analysis in which signals from multiple common SNVs are aggregated into a single test of association for the set of SNVs of interest (see Refs. [1,2] for recent reviews).

Of particular interest in the development of multiple-marker tests is the blurring of the lines that has recently taken place between what were traditionally considered "gene-based" tests of association and "pathway-based" tests of association. Historically, gene-based tests of association aggregated a small number of SNVs within a gene into a single test statistic, whereas pathway-based tests operated in 2 stages [1,2]. In the traditional approach to pathway analysis, researchers first generate a statistic for each gene. In a second stage, researchers combine multiple gene-level statistics into a pathway statistic. Recently, however, a single-stage approach was advocated whereby, in a single stage, all the SNVs in the pathway all simultaneously aggregated into a single statistic [1]. Effectively, this single-stage approach could be considered a "SNV-set" approach where the set of SNVs can be defined in any biologically plausible manner. Although this relatively new approach is gaining in popularity in the literature, little concrete evidence of its performance relative to the 2-stage approach is available. It has been suggested, however, that the 2-stage approach may be optimal when there are fewer causal variants in each gene, but each with strong risk, and the single-stage approach may be better suited for cases where there are more causal variants, but with weaker effects [3-5].

With the rapid growth in access to next-generation sequencing (NGS) technology, there has been a tidal wave of methodological developments to analyze such data. At the heart of most analytic approaches for NGS data is the desire to appropriately handle rare variants. Because statistical tests of individual rare variants lack power and multiple testing penalties quickly become unwieldy because of the preponderance of rare variants, most recently proposed NGS data analysis approaches attempt to aggregate multiple SNV association signals so as to increase power. These rare SNV-set methods can be viewed as a special type of multiple-marker test that is particularly useful when SNVs are rare. For a broad overview and classification of these rare variant approaches for case-control studies see Liu et al [6].

There are few family-based tests of rare variant association that are appropriate for complex pedigrees analyzing dichotomous phenotypes for association with rare variants (see Hainline et al [7] for more extensive discussion). In this article, we apply several of these recently proposed approaches to very large sets of SNVs; the very large sets are created by combining SNVs from multiple genes into pathway based sets of SNVs. The goal of our analysis is to evaluate the appropriateness of these methods for use with massive SNV sets when the proportion of noncausal variants may be very large, but the set may also contain multiple causal variants. We evaluate both single-stage and traditional 2-stage approaches, in which stage 1 computes a test statistic for each gene in the pathway, and stage 2 combines the gene-based statistics into a single statistic for the pathway.

## Methods

### Sample and genes

There were 849 individuals in complex pedigrees with simulated phenotype data available. We classified each of the 849 individuals as either hypertensive (systolic blood pressure >140 mm Hg or diastolic blood pressure >90 mm Hg) or not hypertensive based on whether the individual was classified as hypertensive at any of up to 4 measurements (waves). For our analysis of the simulated data we focused on simulated phenotype 1. We had knowledge of the simulation answers for our analysis. SNVs were mapped to genes using a custom version of ANNOVAR, where a SNV is assigned to a gene if its physical location is within the start-stop position of the gene [8].

### Creation of gene sets

To evaluate the performance of different approaches to the analysis of very large sets of SNVs from multiple genes (eg, pathways) we created 800 sets of 5 genes each, where 200 of the sets contained no genes with causal variants, 200 of the sets contained 1 gene with causal variants, 200 of the sets contained 3 genes with causal variants, and 200 of the sets had all 5 genes containing causal variants. Sets were created by randomly choosing genes, without replacement, from lists of genes that were known (based on the simulation model) to contain or not contain causal SNVs. The average number of SNVs in the 800 sets was 880 (SD = 468; minimum = 58; maximum = 2451).

### Previously proposed statistical tests

We applied 5 different family-based tests of association considered by Zhu and Xiong [9] to sets of SNVs. All tests were conducted in R using software functions written by Zhu and Xiong and custom scripts. More details on the tests are available elsewhere [7,9].

The methods considered by Zhu and Xiong utilize a correction factor, *P_corr_*, which summarizes the additional correlation in the samples that occurs as a result of the complex pedigree structure. *P_corr _*is a function of the estimated kinship matrix (see Hainline et al [7] for details) and is used to adjust the standard error of the test statistics for the additional correlation contained in the pedigree structure.

Zhu and Xiong [9] propose a generalized multivariate *T^2 ^*test comparing the mean allele counts across *n *variants (eg, SNVs within a gene) between the cases and controls; Hotelling's *T^2 ^*test is a multivariate version of the 2-sample t-test. Alternatively, Zhu and Xiong also consider a version that collapses rare variants below a threshold before applying the *T^2 ^*test (combined multivariate and collapsing [CMC]) or uses eigenvectors from the genotype matrix to reduce matrix dimensionality (functional principal component analysis [FPCA]; see Hainline et al [7] for details). In our implementation of CMC we used minor allele frequency cutoffs of 5% and 0.5%. Briefly stated, in all cases, *T^2 ^*is computed as if there was no pedigree structure in the data $\left(T_{initial}^{2}\right)$. The pedigree-adjusted statistic is computed as $T_{initial}^{2}/p_{corr}$. Finally, Zhu and Xiong investigate an approach that applies a single-marker test (pedigree-adjusted single-marker $\chi^{2}$ test of association) to all SNVs in a set, and then uses the minimum *p *value within the set, as the *p *value for the entire set. Thus, there are 5 test statistics in total: *T^2^*, CMC_5%, _CMC_0.5%_, FPCA and $\chi_{min}^{2}$, where we refer to all methods except $\chi_{min}^{2}$ as *T^2 ^*based approaches, because they all rely on the Hotelling's *T^2 ^*statistic.

### Application of the tests to both 1-stage and 2-stage pathway analysis

In our analysis, we applied the 5 methods described above in 2 different ways. First, we directly applied each of the 5 methods to all 800 sets of SNVs (1-stage pathway analysis). All 1-stage pathway analysis tests are of the null hypothesis that no SNVs in the set are associated with the phenotype, with an alternative hypothesis that at least 1 SNV in the set is associated with the phenotype.

Second, we applied each of the 5 methods to all 5 individual genes contained within each of the 800 sets. We then combined the 5 gene *p *values for each test-set combination using Fisher's combined probability test (2-stage pathway analysis). Fisher's combined probability test is defined as $Fisher_{p}=\overset{5}{\underset{i=1}{\sum }}-2\text{log}\left(p_{i}\right)$ where *p_i _*is the *p *value from the *i*^th ^gene in the set and $Fisher_{p}\sim \chi_{10}^{2}$, yielding 1 Fisher's combined probability test *p *value for each of *T^2^*, CMC_5%_, CMC_0.5% _, FPCA, and $\chi_{min}^{2}$, as described in the previous section. Fisher's combined probability test is one of many choices for 2-stage pathway analysis [5], but has the convenient advantage of having a known null distribution when the *p *values being combined are independent (in our case, this means that there is no linkage disequilibrium between the randomly chosen genes in the sets, which is a reasonable assumption). All 2-stage analyses have a null hypothesis that none of the genes in the set of genes contain any SNVs associated with the phenotype, with an alternative hypothesis that at least 1 gene in the set of genes contains at least one SNV associated with the phenotype.

### Modified implementation of the Zhu and Xiong methods

When the Zhu and Xiong approaches are applied to the 800 sets of SNVs, a dramatic inflation of the type I error rate is observed. In particular, among the 5 single-stage approaches, type I error rates ranged from 43.5% to 99.5%, and among the 5 two-stage approaches, type I error rates ranged from 7.5% to 90.5%. Although we do not provide detailed results here, we note that the magnitude of the type I error rate increased as the number of SNVs in the set increased; in particular, the inflated type I error rates only occurred on sets containing more than 200 SNVs. We determined that a potential cause of the inflated type I error rate was in the approach taken when estimating the SNV genotype covariance matrix [10]. When analyzing large sets (>200) of SNVs and given the relatively small sample size (n = 849), prior research shows that the use of the maximum likelihood estimates (MLEs) in covariance matrix estimation may yield unstable results, and that a shrinkage covariance estimator may perform better [10].

We modified the covariance estimation procedure for all methods, replacing the MLEs with the shrinkage estimator. Although we lost the guarantee of the analytic null distribution derived by Zhu and Xiong, we explored the use of the shrinkage estimator using the null distribution in Zhu and Xiong. We found that this approach provided increasingly overconservative results as the set size increased (detailed results not shown). Nonetheless, we still expect that large values of the test statistic should lead to rejection of the null hypothesis.

To account for the effect of set size on the behavior of the statistic, we estimated the empirical cumulative distribution function of the *p *values for the "null" sets (sets containing no causal SNVs) separately as a function of the number of SNVs in the set for each of the test statistics. In particular, $F_{m,n}\left(t\right)=\frac{1}{k}\overset{k}{\underset{i=1}{\sum }}1\left\{p_{i,m}\leq t\right\}$, where *p_i,m_, I = 1...k*, are the *p *values for test statistic *m *(eg, *T^2^*, CMC) when the set size is *n*. To obtain robust estimates of *F(t)*, we binned sets of similar set size; there were 5 bins in total, representing quintile breaks in the null set size distribution. To provide appropriate control of the type I error rate and allow evaluation of the different methods, we computed a modified *p *value, $p{\left(modified\right)}_{i,m}=F_{m,n}\left(p_{i,m}\right)$, for all null and nonnull (contain at least 1 causal SNV) sets. These modified *p *values necessarily control the type I error rate for null sets, allowing us to get a sense of the performance of the different methods if we had an appropriate null distribution for the new test statistics. Although in practice we do not have knowledge of the null set of pathways, this serves as an exploratory analysis to motivate derivation of a closed form null distribution for the modified Zhu and Xiong statistic.

## Results

We applied each of the 10 tests described earlier to all 800 sets of genes. Table 1 illustrates the percent of significant sets (α = 0.05) across the 800 sets, where we stratify the 800 sets into 200 sets containing varying numbers of causal and noncausal genes.

**Table 1:** Percent significant sets at α = 0.05 by number of causal and noncausal genes in the set using modified *p *values

Pathway testing approach	Number of causal and noncausal genes in the set (all sets contain 5 genes)			
	
	**0 causal**, 5 noncausal	**1 causal**, 4 noncausal	**3 causal**, 2 noncausal	**5 causal**, 0 noncausal
Single-stage approach				
FPCA	2.5% (5/200)	6.5% (13/200)	5.0% (10/200)	9.0% (18/200)
$\chi_{min}^{2}$	3.5% (7/200)	6.5% (13/200)	8.5% (17/200)	16.5% (33/200)
*T^2^*	5.0% (10/200)	3.0% (6/200)	1.5% (3/200)	2.5% (5/200)
CMC_5%_	1.5% (3/200)	0.0% (0/200)	2.5% (5/200)	1.5% (3/200)
CMC_0.5% _	3.5% (7/200)	5.5% (11/200)	9.5% (19/200)	8.0% (16/200)
Two-stage approach				
FPCA	2.0% (4/200)	1.5% (3/200)	9.0% (18/200)	5.0% (10/200)
Fishers_$\chi_{min}^{2}$	3.5% (7/200)	4.0% (8/200)	8.5% (17/200)	11.0% (22/100)
Fishers_ *T^2^*	1.5% (3/200)	3.5% (7/200)	2.0% (4/200)	1.0% (2/200)
Fishers_ CMC_5%_	3.0% (6/200)	2.0% (4/200)	3.5% (7/200)	1.0% (2/200)
Fishers_ CMC_0.5% _	2.0% (4/200)	2.0% (4/200)	3.5% (7/200)	0.5% (1/200)

By design, sets containing no causal genes have empirical type I error rates of approximately 5%. The reason for some deviation from 5% in Table 1 is because of a combination of estimating the empirical null distribution based on 200 null sets and the binning procedure used (see Methods). In general, modest increases in the percent of significant sets are observed as more causal genes (genes containing at least 1 causal SNV) are added to the set for single-stage and 2-stage methods based on FPCA and $\chi_{min}^{2}$, single-stage CMC_0.5%_, while other methods showed little difference in the percent of significant null sets and nonnull sets.

## Discussion

Few analyses have considered methods to analyze pathways (sets of genes; large sets of SNVs) for association with rare variants in family studies. Previous research showed that, depending on the underlying genetic architecture, either single-stage or 2-stage approaches to pathway testing may provide a powerful testing approach. However, this result has not been rigorously established across the large class of pathway testing approaches, especially rare variant pedigree data. In this article, we considered both 1-stage and 2-stage approaches to pathway analysis by applying methods proposed by Zhu and Xiong to very large multi-SNV sets (1-stage), or a 2-stage approach using Fisher's combined probability test in conjunction with the methods of Zhu and Xiong.

As with most gene-based testing approaches the authors of the primary methods considered here only evaluated their method on "small" sets of SNVs (189 SNVs) [9]. However, the assumption of many authors of gene-based testing approaches is that the methods can be applied to large sets of SNVs that may contain SNVs from multiple genes (see, eg, Madsen and Browning [3]). This is both a reasonable and natural assumption, although one that has rarely been considered explicitly in the literature.

In our consideration of 1-stage approaches, we found a substantially inflated type I error rate, which increased as the number of SNVs increased. Further analysis pinpointed the issue to the use of MLEs when estimating the genotype covariance matrix (as in the Zhu and Xiong code). Schafer and Strimmer [10] showed that a shrinkage estimator provides robust covariance estimates as the number of SNVs increases relative to the sample size. When we implemented the shrinkage estimator, the results were overly conservative (empirical type I error rate was substantially less than the nominal rate) when using the null distribution for MLEs from Zhu and Xiong. This pattern of findings also held true for 2-stage approaches when any genes in the set were large (containing more than 200 SNVs). To address these limitations, we applied an empirical correction factor to the *p *values, which allowed us to examine performance of the 10 methods.

## Conclusions

Further research is necessary to develop alternative covariance estimation procedures and corresponding null distributions for large (more than 200 SNVs) sets. We note that, in both a companion paper [7] and here, use of the MLE estimation approach yields well-controlled type I error rates for SNV sets with less than 200 SNVs. Permutation approaches to control type I error should also be explored, however, given the complex pedigree structure present in the data will require gene dropping or related approaches that may limit their practical utility.

Assuming that appropriate finite sample null distributions can be derived when applying SNV-set methods to very large sets of SNVs, the true underlying genetic disease architecture will play a significant role in determining which statistical methods will perform best in practice. A recent article evaluating the differences in rare variant tests of association illustrated that for sets of SNVs where the proportion of noncausal SNVs is very large, as will likely be the case in 1-stage approaches for rare variants, tests like the minimum $\chi_{min}^{2}$ (equivalent to an L_∞ _norm) will perform better than *T^2^*-based (L_2 _or L_1 _norm) type tests (see Liu et al [6] for details) by empirically upweighting the strongest SNV-phenotype associations.

Few methods exist for the analysis of potential relationships between binary phenotypes and rare genetic variation; fewer still have considered how such methods will perform on very large sets of SNVs that may span multiple genes. Our analysis identified substantial inflation of the type I error rate using a standard approach, and so we implemented an empirical approach to evaluate the relative performance of different methods. Further research is necessary to explore robust test statistics and analytic strategies for large SNV sets in complex pedigrees across a wide variety of genetic architectures.

## Competing interests

The authors declare that they have no competing interests.

## Authors' contributions

All authors participated in design of the overall study; AL and AB carried out various aspects of data preprocessing, including gene mapping and building preliminary data analysis files; BG estimated the kinship matrices; CA and AH implemented the 4 FBAT tests; AL, BG, and NT ran statistical tests, analyzed data, and conducted literature review. AL investigated a novel statistic; NT drafted the final manuscript. All authors read and approved the final manuscript.

## References

[B1] Petersen A Spratt J Tintle NL Gondro C, van der Werf J, Hayes B Incorporating prior knowledge to increase the power of genome-wide association studies Genome-Wide Association Studies and Genomics Prediction 2013 New York, Springer 519 541 10.1007/978-1-62703-447-0_25 23756909

[B2] Petersen A Alvarez C DeClaire S Tintle NL Assessing methods for assigning SNPs to genes in gene-based tests of association using common variants PLoS One 2013 8 e62161 10.1371/journal.pone.0062161 23741293 PMC3669368

[B3] Madsen BE Browning SR A group-wise association test for rare mutations using a weighted sum statistic PLoS Genet 2009 5 e1000384 10.1371/journal.pgen.1000384 19214210 PMC2633048

[B4] Petersen A Sitarik A Luedtke A Powers S Bekmetjev A Tintle NL Evaluating methods for combining rare variant data in pathway-based tests of genetic association BMC Proc 2011 5 S48 10.1186/1753-6561-5-S9-S48 22373429 PMC3287885

[B5] Fridley BL Biernacka Gene set analysis of SNP data: benefits, challenges and future directions Eur J Hum Genet 2011 19 837 843 10.1038/ejhg.2011.57 21487444 PMC3172936

[B6] Liu K Fast S Zawistowski M Tintle NL A geometric framework for evaluating rare variant tests of association Genet Epidemiol 2013 37 345 357 10.1002/gepi.21722 23526307 PMC3718063

[B7] Hainline A Alvarez C Luedtke A Greco B Beck A Tintle NL Evaluation of power and type I error of recently proposed family-based tests of association for rare variants BMC Proc 2014 8 suppl 2 S36 10.1186/1753-6561-8-S1-S36 PMC4143711 25519321

[B8] ANNOVAR: Functional annotation of genetic variants from high-throughput sequencing data -http://www.openbioinformatics.org/annovar 10.1093/nar/gkq603 PMC2938201 20601685

[B9] Zhu Y Xiong M Family-based association studies for next-generation sequencing Am J Hum Genet 2012 90 1028 1045 10.1016/j.ajhg.2012.04.022 22682329 PMC3370281

